# GVHD Pathogenesis, Prevention and Treatment: Lessons From Humanized Mouse Transplant Models

**DOI:** 10.3389/fimmu.2021.723544

**Published:** 2021-07-29

**Authors:** Nicholas J. Hess, Matthew E. Brown, Christian M. Capitini

**Affiliations:** ^1^Department of Pediatrics, University of Wisconsin School of Medicine and Public Health, Madison, WI, United States; ^2^Department of Surgery, University of Wisconsin School of Medicine and Public Health, Madison, WI, United States; ^3^University of Wisconsin Carbone Cancer Center, Madison, WI, United States

**Keywords:** graft-versus host disease, xenogeneic transplantation, humanized mouse models, hematopoietic stem cell transplantation, T cells

## Abstract

Graft-*vs*-host disease (GVHD) is the most common cause of non-relapse mortality following allogeneic hematopoietic stem cell transplantation (HSCT) despite advances in conditioning regimens, HLA genotyping and immune suppression. While murine studies have yielded important insights into the cellular responses of GVHD, differences between murine and human biology has hindered the translation of novel therapies into the clinic. Recently, the field has expanded the ability to investigate primary human T cell responses through the transplantation of human T cells into immunodeficient mice. These xenogeneic HSCT models benefit from the human T cell receptors, CD4 and CD8 proteins having cross-reactivity to murine MHC in addition to several cytokines and co-stimulatory proteins. This has allowed for the direct assessment of key factors in GVHD pathogenesis to be investigated prior to entering clinical trials. In this review, we will summarize the current state of clinical GVHD research and discuss how xenogeneic HSCT models will aid in advancing the current pipeline of novel GVHD prophylaxis therapies into the clinic.

## Introduction

Since the first successful allogeneic hematopoietic stem cell transplant (HSCT) was performed in 1956 by E. Donnall Thomas, its use has grown exponentially (1). While allogeneic HSCT is a curative approach for many malignant and non-malignant diseases, a majority of patients will develop life-threatening complications highlighted by graft-*vs*-host disease (GVHD) or relapse (in the malignant disease setting) within three years post-transplant (2, 3). GVHD is defined by the recognition and reactivity of the donor immune cells for recipient antigens (alloreactivity) that eventually leads to organ-specific pathologies to develop (classically the skin, gastrointestinal tract and liver). Importantly, the balance between too much and too little alloreactivity if often what determines a patients probability of developing GVHD (too much) or relapse (too little) (4, 5). As such, the ability to predict and control the graft-*vs*-host (GVH) response underscores the highly complicated goal for HSCT research (6).

The application of allogeneic HSCT as the first true immunotherapy was not fully appreciated until T cell-depleted (TCD) grafts were investigated as a means to eliminate GVHD (7, 8). While TCD grafts were successful in decreasing GVHD to extremely low frequencies, TCD grafts were also associated with unacceptable rates of infections, poor engraftment, Epstein Barr virus (EBV)-reactivation-induced lymphoproliferative disease and elevated rates of relapse (8, 9). From this observation, the field began to acknowledge the novel graft-*vs*-leukemia (GVL) activity the donor cells have in controlling malignant disease. While αβ T cells (which will be the main focus of this review) have been the primary focus of many studies, ongoing studies are also exploring the role of NK cells and γδ T cells as donor-lymphocyte infusions (DLI) to treat/prevent relapse post-HSCT (NCT01823198, NCT01904136, NCT03533816) (9, 10). The application of these cell populations in DLI is extremely exciting because they are naturally cytotoxic and do not cause GVHD, though their inability to form memory responses remains a major hurdle for long-term disease surveillance.

Overall relapse rates of patients undergoing allogeneic HSCT have remained fairly unchanged in the past few decades. However, multiple advancements have been made including: improved human leukocyte antigen (HLA) class I/II genotyping leading to more precise HLA compatible grafts (11), the establishment of reduced-intensity and non-myeloablative conditioning regimens for older patients (12), the introduction of alternative graft sources (G-CSF mobilized peripheral blood and umbilical cord blood) (13, 14), novel T-cell specific prophylaxis drugs (15, 16) and most recently, the widespread use of post-transplant cyclophosphamide as a method of *in vivo* allo-reactive T cell depletion (17, 18) have all significantly improved allogeneic HSCT outcomes.

Research into the fundamental mechanisms of T cell activation following allogeneic HSCT have also advanced tremendously in the past few decades thanks to the genetic tractability and feasibility of using murine models. Through these means, the field has identified numerous pathways/targets that contribute to the GVH reaction with many currently being studied in clinical trials. Unfortunately though, many of these targets will not translate into the clinic; while a less-than-perfect success rate in clinical trials is to be expected, many of these failures are most likely a result of fundamental differences in murine and human immunology (19). Thus, there remains an “open niche” in the field for a mouse-to-human translational model system to help identify and triage targets for clinical trials.

In this review, we will highlight research investigating GVHD using humanized mouse models and discuss how the growing use of humanized mice have the potential to revolutionize the field. To do this, we will highlight each signal of the three-signal hypothesis of T cell activation (T cell receptor, cytokines and co-stimulation) individually and contrast the relative insights each model system (murine, humanized and clinical) has made toward understanding how each signal impacts the development and pathology of GVHD.

## Humanized Mice for GVHD Research

With the development of the NSG (NOD.Cg-*Prkdc^scid^ Il2rg^tm1Wjl^*/SzJ) mouse in 2005 by Leonard Shultz, it became possible to transplant human immune cells and/or immune-progenitors into these mice to study aspects of human immunology/hematopoiesis (20). Shortly, these mice allow for human immune cell persistence due to several key mutations including: a NOD background specific SIRPα mutation that allows binding to human CD47 to prevent phagocytosis; a SCID mutation that prevents T/B cell development; and a null IL2Rγ (common γ chain or CD132) mutation that prevents signaling from cytokine receptors utilizing the IL2Rγ chain (which includes IL-2, IL-4, IL-7, IL-9, IL-13, IL-15 and IL-21) (20). Overall, these mutations result in a lymphopenic mouse that lacks T/B cells (due to the SCID mutation) and NK cells (due to the lack of IL-15 signaling required for NK cell development) which were the primary mediators of human cell rejection after xenotransplantation (20). Alternatively, while the myeloid, granulocytic and non-hematopoietic cells (e.g. endothelial and stromal cells) compartments of NSG mice are “blind” to the presence of human cells (due to the SIRPα mutation), they are still present at similar frequencies as wild-type BALB/c mice and are fully capable of sensing and responding to damage-associated-molecular-patterns (DAMPs). Furthermore, these remaining cell populations remain essential components of xenogeneic transplants through their ability to present host murine antigens (Signal 1) and produce inflammatory cytokines (Signal 3).

Since the creation of the NSG mouse, several additional “next-generation” immune-deficient mice capable of humanization have been developed including the NSG-SGM3, MISTRG, NBSGW, NOG, NRG and NSG-HLA-A2 mice (21–26). Importantly, the term “humanized” has sometimes become synonymous with human cell “engraftment”, with the latter term generally reserved for model systems studying human hematopoiesis or *de novo* generation of human immune lineages that are capable of self-renewal and long-lasting human cell immune reconstitution. To better distinguish these studies from human transgene-alone (e.g., HLA-A2, hACE2) without human cell/tissue transplantation, model systems incorporating human immune cells engrafted into these host strains are now referred to as “human immune system” or “HIS” mice.

Alternatively, immune-deficient mice can also be used to study the human T cell response to xenogeneic antigens. These studies are often much shorter in duration, with the outcome either death due to GVHD or short/moderate persistence of human T cells that are not *de novo* generation. Peripheral blood-humanized mice (PBL-Hu) are commonly used for these studies for these studies though modern versions also utilize isolated T cells or T cell subsets from primary human HSCT graft tissue including bone marrow, G-CSF mobilized peripheral blood or umbilical cord blood. Additionally, human cancer cell lines and patient-derived cancers (commonly referred to patient-derived-xenograft or PDX models) can be transplanted into immunodeficient mice prior to human graft tissue to examine the GVL effect. In this review, we will describe these transplant-related versions of humanized mice research with the general term of “xenogeneic transplant” model systems.

## The TCR : MHC Interaction: Signal 1

Of the three T cell activation signals, signal 1 remains arguably the most important and mandatory for successful activation. The strength of any T cell response is due in part to the diversity of the T cell receptor (TCR) repertoire and their ability to recognize non-self-antigens (27–29). Unlike current immunotherapies that target one or two antigens (i.e. monoclonal antibodies, CAR-T cells and BiTES), the T cell response after an allogeneic HSCT has the capacity to target tens to hundreds of different antigens that prevent the cancer from escaping though antigenic escape and allow for long-term prevention of relapse (30, 31). Unfortunately, this phenomenon is not limited to cancer-associated-antigens with T cell responses against host-antigens often developing into GVHD (32). This section is dedicated to understanding how antigenic targeting by the TCR can instruct both the GVL and GVH responses.

### Different T Cell Populations Influence GVHD Development

T cells are broadly divided along two separate lineages; the CD4 *versus* CD8 lineage represent modulatory and cytotoxic functions respectively; and the naïve (CD45RA) *versus* memory (CD45RO) lineages denoting antigen-inexperienced or -experienced respectively. While there are numerous other sub-populations of T cells (some of which will discussed in the “Extracellular Messengers: Signal 3” section), this review will focus on the T cell lineages highlighted above.

In murine models of allogeneic HSCT, two independent groups have shown that murine memory T cells are not able to mediate GVHD (33–35). These groups theorized that since memory T cells are already antigen-experienced, there is a low likelihood of them having additional cross-reactivity with a host allo-antigen. Cross reactivity of memory T cells to allo-antigen though has been detected when viral-specific memory T cells were cultured with mismatched HLA molecules, though these studies also highlighted that these viral-specific memory T cells did not cause GVHD in a cohort of 153 patients, 73 of which had an HLA mismatch (36, 37). This may be due to a suboptimal TCR signal of the cross-reactive HLA leading to anergy or an abortive T cell response.

Two recent phase I studies have transitioned this work to investigate naïve T cell depleted or CD8^+^ memory T cells for donor-lymphocyte infusions (DLI) respectively (38, 39). Both of these studies showed that DLI infusions with their respective T cell populations were safe, feasible and were associated with a low incidence of acute GVHD (aGVHD). Despite these observations, a recent phase II study analyzing the usage of naïve T cell depleted grafts compared to historical controls showed no difference in grade II-IV aGVHD. One limitation of this study though is that the naïve T cell depleted arm received calcineurin inhibitor (CNI) monotherapy for aGVHD prophylaxis (compared to CNI plus methotrexate) and a more myeloablative conditioning regimen than the historical controls. The study also reported that only 3/35 patients developed grade III aGVHD and all patients were steroid-responsive (40). There was no difference in engraftment rates or EBV/cytomegalovirus (CMV) reactivation showing that naïve T cell depleted grafts do not suffer from the same complications as T cell depleted grafts (9). The use of naïve T cell depleted grafts is also currently under investigation in a phase II trial comparing four different GVHD prophylaxis regimens (NCT03970096).

In regard to the role that CD4 and CD8 T cell lineages have in GVHD, very few clinical studies have investigated this directly. One randomized double-blind phase II study performed in 1994 selectively depleted CD8 T cells from 19 bone marrow grafts transplanted into HLA-identical sibling donors with CNI monotherapy for GVHD prophylaxis. The overall incidence of grade II-IV in the CD8-depleted arm was 20% and 80% in the 17 control patients (41). While this study highlights the importance of the CD8 lineage in GVHD pathogenesis, to our knowledge no further studies have followed up on this observation.

### Human T Cell Reactivity After Xenogeneic Transplantation

**Figure 1:**
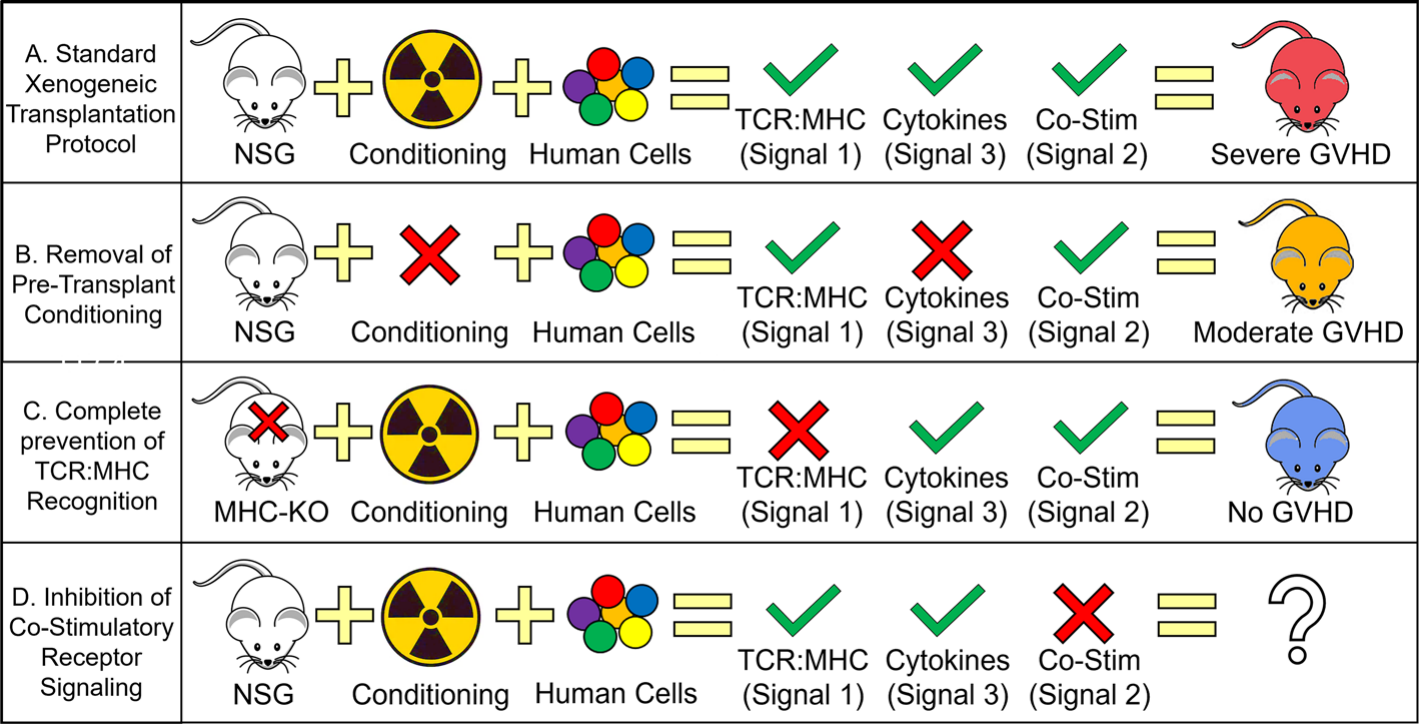
Human T cell Requirements for GVHD Development During Xenogeneic Transplantation. Schematic depicting the relative contribution of each T cell activation signal toward the development of GVHD. **(A)** Standard xenogeneic transplant protocols provide all three T cell activation signals, human TCR to murine MHC recognition, pro-inflammatory cytokine secretion from genotoxic conditioning (i.e. γ-irradiation) and human CD28 to murine B7 cross-reactivity (with possible contributions from other co-stimulatory proteins) to cause severe GVHD. **(B)** Removing the presence of pro-inflammatory cytokines by not conditioning NSG mice prior to transplant results in only a slight decrease in GVHD severity with clinical data using tocilizumab/ruxolitinib also showing modest effects on GVHD mitigation. **(C)** Complete prevention of human TCR recognition of murine MHC (by knocking out murine MHC) eliminates all signs of GVHD. The widespread adoption of calcineurin inhibitors (e.g. tacrolimus) for GVHD prophylaxis also supports the important role of TCR : MHC interactions though in the case of clinical calcineurin inhibitors, only a partial inhibition is achieved. **(D)** Blocking co-stimulatory signaling remains the only T cell activation signal not investigated with xenogeneic transplant studies and is only recently entered the clinical domain. Severe GVHD is generally described as achieving ≥ 70% lethality with 3E^6^ PB-MNC with moderate GVHD ranging from 30-70% lethality with the same dose of human cells.

With the clinical observations noted above, one question was if transplantation of human cells into NSG mice (xenogeneic transplantation) can model these same T cell responses. Initially, it was unknown whether human TCRs could even recognize murine major histocompatibility complex (MHC) complexes and if they did, if the result be a GVHD-like disease (20, 42). In an elegant study, Brehm et al. showed that human T cells transplanted into γ-irradiated NSG mice developed acute signs of GVHD that included liver, lung and skin pathology followed by extreme weight loss and death. Furthermore, they showed that human T cells transplanted into NSG mice lacking both MHC class I and II expression did not develop GVHD, persisted and were able to reject an allograft of human islet cells (43). In another study, the use of CNI was able to ablate xenogeneic GVHD development (44). These studies confirm that human TCR can recognize both murine class-I and -II MHC and that a successful TCR signal is required to initiate a successful GVH (i.e. xenogeneic) response (20, 42–44) (**Table 1** and **Figure 1**).

**Table 1:** List of mouse to human cross-reactive molecules.

Murine Component	Human Component	Cross- Reactivity	Reference
TCRComplex			
MHC Class I	TCR	Yes	43
MHC Class I	CD8	Yes	46
MHC Class II	TCR	Yes	43
MHC Class II	CD4	Yes	45
Cytokine Receptors			
IL-2	IL-2R	Yes*	56
IL-3	IL-3R	No	21-26
IL-4	IL-4R	No	56, 85
IL-6	IL-6R	No	86
IL-7	IL-7R	Yes	54
IL-10	IL10-RA	?	n/a
IL-12	IL12R	Yes	87-88
IL-15	IL-15R	Yes	56
IL-17A	IL-17R	?	n/a
IL-23	IL23R	Yes	87-88
IFNα/β	IFNAR	No	99-100
IFNy	IFNGR	No	99-100
Type Ill Interferons	IFNLR1/IL10RB	Yes	101
M-CSF (CSF1)	M-CSFR (CD115)	No	21-26
GM-CSF (CSF2)	GM-CSFR (CD116)	No	21-26
G-CSF (CSF3)	G-CSFR (CD114)	Yes	21-26
TNFα	CD120a	Yes	21-26
FLT3L	FLT3	Yes	21-26
TGF-β	TGF-βR1-3	Yes	21-26
SCF	CD117	Yes	20
SDF-1	CXCR4	Yes	20
TNF Receptor Superfamily (TNFRSF)			
OX40L (CD252)	OX40 (CD134)	?	n/a
FASL (CD178)	FAS (CD95)	?	n/a
CD70	CD27	?	n/a
4-1BBL (CD137L)	4-1BB (CD137)	?	n/a
CD40	CD40L (CD154)	?	n/a
Immunoglobulin Superfamily (lgSF			
B7 (CD80/86)	CD28	Yes	128
B7 (CD80/86)	CTLA-4 (CD152)	Yes	128
PD-L1 (CD274), PD- L2 (CD273)	PD-1 (CD279)	?	n/a
ICOSL (CD275)	ICOS (CD278)	?	n/a

The other constituent of the human TCR to murine MHC complex are the human CD4 and CD8 molecules responsible for binding and stabilizing the TCR : MHC interaction. In one study, researchers showed that insertion of the human CD4 gene into mice deficient in murine CD4 was sufficient to restore the murine CD4 population (45). In a separate study, another group using biochemical analyses showed that human CD8 can bind to murine H2K^b^, initiate killing of cells infected with a target antigen and that this interaction can be blocked with a CD8 antibody (46). These group of studies support the hypothesis that the human TCR complex is compatible with murine MHC to elicit antigen-specific immune responses (**Table 1** and **Figure 1**).

Next, it was determined that T cells can become xenoreactive after transplantation. Several studies have shown that human T cells develop into an effector memory population (CD45RO^+^, CD27^+^, CCR7^-^, CD62L^-^) shortly after transplantation with very few naïve (CD45RA^+^) T cells detected (47, 48). Importantly, most of these studies were conducted with primary human peripheral blood that contains very few hematopoietic stem and progenitor cells (HSPCs) such that *de novo* T cell generation cannot occur. Additionally, the thymus of NSG mice atrophies shortly after birth and is completely absent by 4-6 weeks of age, negating the likelihood of *de novo* T cell production in these model systems. The same effector memory phenotype has also been identified in several primary human T cells clones taken from GVHD patients (49–52). While there has not been a study directly investigating the capacity of isolated human memory T cells to mediate GVHD in a xenogeneic transplant model, studies using human umbilical cord blood T cells (which are all naïve CD45RA^+^ T cells) also detect a universal transition to an effector memory phenotype several weeks after transplantation (47).

Interestingly though, this same effector memory transition was detected when human T cells were transplanted into MHC class-I and -II deficient mice, who did not develop GVHD (43). This suggests that this phenotype is not solely antigen-driven and may in fact be caused by homeostatic proliferation (53). Homeostatic proliferation arises when T cells are transplanted into a lympho-deplete environment high in IL-2 and IL-7, which occurs in HSCT patients and in NSG mice. Though in the latter case, murine IL-2 requires 5-10 times the concentration for equivalent activation of human T cells while murine IL-7 is fully cross-reactive (54–56). The potential mechanisms and consequences of homeostatic proliferation in immunodeficient mice have been reviewed previously (53). While an effector memory phenotype is associated with GVHD in xenogeneic mice and clinical GVHD samples, the cause of this phenotype may most likely be homeostatic proliferation and thus not a valid marker of alloreactive (or xenoreactive) T cells.

Lastly, one xenogeneic transplant study has investigated the role of CD4 and CD8 T cells in GVHD. This study showed that isolated CD8 T cells but not CD4 T cells were necessary for xenogeneic GVHD (44). While we await further studies dedicated to exploring the specific pathologies and activation pathways used by human CD4 versus CD8 T cells in xenogeneic transplantation, these limited but highly interesting studies suggest that CD8 T cells and not CD4 T cells may be the more prominent T cell lineage to study in terms of GVHD pathology.

### The Clonal Response to Xeno-Reactive Antigens

Pioneering studies on the role of antigen-presenting-cells (APCs) in GVHD development have shown that host hematopoietic and non-hematopoietic APCs are responsible for alloreactive antigen presentation (57–60). This was further confirmed in two xenogeneic transplant studies that used isolated human T cells. These studies showed that even in the absence of human (donor) APCs, GVHD still occurs at similar frequencies as in unmanipulated human grafts (43, 47). Additionally, when γ-irradiated NSG mice are used for xenogeneic transplantation, they develop GVHD almost universally. In contrast, when non-irradiated NSG mice are used, GVHD is less prevalent/delayed and highly manipulatable based on the cell dose, graft tissue, graft composition and the host inflammatory status (see Extracellular Messengers: Signal 3 for further discussion) (47). These observations highlight several unique possibilities in terms of the specific antigenic stimulation experienced by donor T cells.

Antigenic stimulation in T cell is generally described by the type of HLA mismatching that occurs between the donor and host. MHC mismatches occur due to a complete mismatch of the HLA allele. In murine models, this is often either a C57BL/6 or BALB.B strain (both express H-2^b^) into a B10.BR (H-2^k^), C3H (H-2^k^) or BALB/c (H-2^d^) strain. In the clinic, this occurs when there is a defined HLA mismatch at one or more of the HLA loci (see “The Importance of HLA Matching”). Minor histocompatibility mismatches occur between donor and host despite matching HLA loci and are thought to be caused from variations within individual HLA loci (i.e. allelic diversity in humans) and from the expression of non-classical HLA peptides. While the T cell reactive antigen in minor histocompatibility mismatches is almost certainly due to variations in the presented peptide structure, it is unclear if the primary antigen in MHC mismatches is the mismatched HLA peptide itself or the unique repertoire of peptides it can present compared to the host genotype.

In the case of xenogeneic transplantation studies, NSG mice express the H2^g7^ haplotype consisting of HLA-K^d^, -D^b^, IA^g7^, IE^null^ which would represent MHC mismatches at three different loci (two MHC class I and one MHC class II locus) (20). Assuming NSG mice express the same repertoire of murine antigens, one hypothesis would be that a similar TCR clonality would develop post-xenotransplantation. In one study, investigators showed that TCR diversity was indeed reduced 14 days after xenotransplantation when compared to the initial sample, but it remained surprisingly diverse overall (48). Interestingly though, there was a very low overlap between CD4^+^ and CD8^+^ T cell clonotypes shared between NSG mice receiving the same donor graft which prevented the authors from correlating specific clonotypes with GVHD. The authors surmised that this may be due to the presence of xeno-reactive T cells existing at a low frequency in the starting human peripheral blood graft (48). This may also help explain the observation from another study that showed an LD_50_ from GVHD after transplantation into non-conditioned NSG mice of 3E^6^ peripheral blood mononuclear cells (47). If xeno-reactive T cell clones are indeed rare, this may explain the variability in lethal GVHD seen even when the same human graft tissue is transplanted into identical NSG mice as each mouse could receive a different set of human T cell clones. It has been approximated that ~1E^11^ T cell circulate through a human body with ~1E^9^ unique clonotypes present (61). While this is much lower than the estimated maximum number of clonotypes that could exist (~1E^15^), it is also much higher than in mice, thought to be around 2E^6^ different clonotypes (61).

Despite the difficulty in overcoming the diversity of the human T cell clonality, two studies (one in humans and the other in mice) suggest that the field may be able to elucidate xeno-reactive (or allo-reactive) T cell clones in the future. One study investigated the TCRβ repertoire from 15 different allogeneic HSCT patients with various degrees of HLA mismatching (62). All patients were diagnosed with gastrointestinal tract (GI) GVHD with the cohort further divided by those having steroid-refractory GVHD (SR-GVHD) and those that were steroid-responsive. They reported that although each patient had a unique TCRβ clonal structure with little overlap between patients, SR-GVHD patients had a more conserved TCRβ clonality that steroid-responsive patients (62). Furthermore, they showed that over time, the same T cell clones identified in the GI tract expanded in the blood of SR-GVHD patients but not the steroid-responsive patients (62). A separate study performed in mice revealed that the T cell clonality in the GI tract after transplant was dependent on the host mouse strain (63). The authors took C57BL/6 graft tissue and performed syngeneic (into C57BL/6), minor histocompatibility mismatch (into BALB.B) or two different MHC mismatch transplants (B10.BR and BALB/c). In each case, the resulting T cell clonality was different among each host strain with the authors able to predict the recipient mouse strain based on the overall clonal architecture (63). While it remains unclear if the dominant antigen in xenogeneic transplantation (or MHC mismatches in general) is directed against the specific mismatched HLA peptide or the antigens it presents, these studies highlight the possibility of using TCRβ diversity (i.e. Vβ spectratyping and/or TCRB sequencing) as a measurement of GVHD responses (64).

### The Importance of HLA Matching

T cell development in the thymus is a carefully orchestrated process to ensure that mature T cells have HLA affinity (positive selection) while minimizing reactivity to host antigens (negative selection). As a result, each individual’s TCR repertoire at any given time reflects their own unique HLA genotype. For example, due to the diversity of alleles and variation within those alleles, other than identical twins, it is unlikely more than 10 to 100 people in the world express the exact same immunopeptidome and thus have the exact same clonal TCR architecture though the presence of public TCRs (shared TCR clones between individuals) still occurs quite frequently (27–29).

This highlights the importance of having high quality HLA-matching for allogeneic HSCT, as even a minor variation in HLA could introduce a multitude of alloreactive antigens capable of being recognized by the donor T cell population. Currently, the standard of care is 8/8 allele matching (HLA-A, -B, -C and -DRB1) with 10/10 matches (which include HLA-DQ) becoming increasingly common (11). Additionally, our knowledge of permissive and non-permissive HLA-DPB1 alleles, which is dependent on the relative overlap of the immunopeptidome continues to grow (65, 66). Although genotyping is becoming increasingly sensitive to allelic variation, GVHD can still develop in 10/10 matched unrelated donors suggesting that a deeper understanding of the mismatches in HLA class Ib alleles (HLA-E, -F, -G and -H), HLA-DM (despite their relatively low expression and allelic diversity) and millions of minor histocompatibility antigens (e.g. H-Y) may be necessary to fully understand a patient’s susceptibility/probability of developing GVHD (11).

Over the years though, there have been several studies that have isolated specific alloreactive T cell clones from GVHD patients. Expansion of these clones, primarily from the skin or blood, against host cells revealed HLA-restricted cytotoxic CD4^+^ and CD8^+^ T cell clones (49–51). Furthermore, the TCRα/β usage was extremely diverse across patients and even in clones directed against the same HLA allele within an individual patient (49). The clones isolated in these studies were shown to target HLA-A, -B and HLA-DR, -DQ and -DP mismatches (49–51, 67). While the majority of these studies were completed before 2000 and did not have the capabilities of modern-day sequencing technology, they nevertheless highlight the capacity of GVH responses to develop against mismatched HLA alleles.

### Organ Specificity in GVHD

In the clinic, aGVHD manifests primarily with gastrointestinal tract, skin and/or liver pathogenesis (32). In xenogeneic transplant models, the same repertoire of organs are affected in addition the lungs which in the clinic is normally restricted to chronic GVHD pathology (20, 43, 47, 48). While most of the same organs are affected, the severity and prevalence of each organ’s pathology is altered. Liver, lung and gastrointestinal tract pathology is common with the skin having variable responses (in contrast to skin being the most common organ affected in the clinic) (20, 43, 47, 48). Additionally, when NSG mice are not conditioned prior to transplant, there is no gastrointestinal tract pathology observed (47).

The idea that GVHD is an organ-specific disease is currently under investigation and remains unresolved. Using an MHC-matched, minor histocompatibility mismatched murine model with 2-photon microscopy, one study suggested that CD4 and CD8 T cells were relatively stationary in GVHD target organs, with few T cells entering or egressing out of the tissues after initial pathology was established. This study also showed that tissue residency of the T cells was dependent on the direct interactions with tissue-resident APCs (68). In contrast, another study in rhesus macaques used serial intravascular staining and scRNA-seq to show that alloreactive T cells were identifiable in the blood and developed a transcriptional signature of tissue invasiveness (i.e. ITGB2, CD74 and others). They surmised that alloreactivity may develop in the circulation/lymph system before tissue residency is established, though the timing and the site of initial T cell activation are still to be fully supported (69).

## Extracellular Messengers: Signal 3

While signal 2 (co-stimulatory proteins) would classically be discussed next, there are substantially more basic and clinical studies investigating the role of cytokines in GVHD. As such, this review will follow a similar path as the field and discuss the role signal 3 has on GVHD before signal 2.

Cytokines are often deemed accessory to optimal T cell activation, though it is clear that they play important roles in directing and shaping the T cell immune response (70, 71). They also represent systemic mediators of inflammation capable of interacting with almost every organ in the body (70). Due to their relative abundance in the circulation, they have been much easier to study and as a result, have been and remain at the forefront of GVHD prophylaxis research.

### Influence of Conditioning on GVH Responses

The role of conditioning regimens on the outcomes of HSCT have changed drastically since the first bone marrow transplantation (12). HSCT was originally designed to “rescue” a patient’s immune system after an otherwise lethal dose of irradiation and/or chemotherapy. High dose irradiation/chemotherapy was given to eliminate residual leukemia from the body but had the side-effect (among others) of destroying the patients HSPCs (1). While high dose irradiation/chemotherapy, now called myeloablative conditioning (MAC), is still used today, the field has generally trended toward the use of lesser (less damaging) forms of MAC conditioning, thanks in part to the observation that there is substantial anti-leukemic activity from donor cells (31, 72).

The use of both reduced intensity (RIC) and non-myeloablative conditioning (NMA) regimens has expanded the use of allogeneic HSCT to older patients who otherwise would not of survived MAC and children with non-malignant disease who do not require such intensity from their conditioning regimen (12). Additionally, the intensity of conditioning regimen is directly correlated with relapse and GVHD rates in patients. NMA/RIC regimens have higher rates of relapse but decreased frequencies of GVHD when compared broadly (6, 12). As such, no specific conditioning regimen has emerged superior to another, with most clinics operating on patient or disease specific criteria as to which conditioning regimen a patient receives. These observations though highlight two questions; what is the modern-day purpose of conditioning and is conditioning required for GVHD to develop?

In regard to the role of conditioning in GVHD development, there are two metrics that are strongly associated with GVHD development. The first is not directly related to conditioning but involves the mismatching of the donor/recipient HLA (see the Importance of HLA Matching above) in which case mismatched transplants generally receive a harsher conditioning regimen to facilitate engraftment. The second is the degree of host damage which is also associated with a more myeloablative conditioning (12). This is highlighted by the MAGIC consortium that have used the serum biomarkers sST2 and REG3α, both released by damage host cells, as predictors of non-relapse mortality (NRM) and SR-GVHD (73, 74). In addition to sST2 and REG3α, necrotic and pyroptotic (two inflammatory forms of cell death) host cells release a variety of damage-associated-molecular-patterns (DAMPs) that activate the innate immune response (e.g. ATP, mtDNA, HSPs and HMGB1) leading to a cytokine storm of pro-inflammatory cytokines (e.g. IL-1α/β, IL-6, IL-8 and IL-12) (70, 71). As a result, most of the prospective GVHD prophylaxis clinical trials have focused on these cytokines to prevent GVHD development. Interestingly though, several xenogeneic transplant studies have shown that GVHD develops irrespective of conditioning the host (via γ-irradiation) though at a much reduced frequency (21, 47). Furthermore, the penetrance of GVHD can be modified with the addition of LPS to mimic the inflammatory environment post-conditioning (47). These early xenogeneic studies suggest that inflammatory cytokines have a modulatory role in shaping the GVHD response but may not be required for its development.

Now that the graft-*vs*-leukemia (GVL) effect by donor graft tissue is well accepted, do allogeneic HSCTs require conditioning? Conditioning serves two purposes beyond that of killing residual leukemia cells (and non-leukemic cells). The first is to remove the host HSPCs. Both MAC and RIC regimens are sufficient to eliminate the host HSPCs while NMA do not offer complete elimination of host HSPCs (12). As a result, NMA regimens often suffer from mixed chimerism and may require a DLI to maintain donor engraftment (12). The second purpose is to suppress/deplete enough of the host immune cells that the donor cells (specifically the HSPCs) are eliminated before engraftment in the bone marrow can occur (12). As a result, there has recently been a movement in the field to develop targeted and/or non-genotoxic conditioning regimens (75).

To our knowledge, there are currently six different antibody/antibody-drug-conjugate (ADC) based conditioning regimens in development. All of these candidates use either the immune cell marker CD45 or the HSPC-specific marker CD117 to target immune cells for clearance, sparing non-immune cells from any off-target damage. Of the two CD117 antibody-based conditioning regimens, JSP191 and MGTA-117, JSP191 has progressed the furthest so far. JSP191, formerly known as AMG191 or SR-1, has had extensive pre-clinical studies performed showing its ability to deplete both mouse and human HSPCs in a dose-dependent manner that allows for adoptive transfer of allogeneic HSPCs (76–79). JSP191 is now currently in a phase I clinical trial for use in severe-combined immunodeficiency disorder (SCID) patients prior to transplant (NCT02963064). MGTA-117 is a CD117 antibody conjugated to amanitin, a potent inhibitor of RNA polymerase II/III, that plans on starting a phase I/II clinical trial in relapse/refractory AML/MDS patients in late 2021. The four CD45 antibody based conditioning regimens are conjugated to either iodine^131^ (β/γ emitter), astatine^211^ (α emitter), yttrium^90^ (β emitter) or saporin (non-radioactive) (80, 81). Of these, the CD45-iodine^131^ candidate is part of the IOMAB-B phase III clinical trial investigating its use in older AML patients followed by NMA conditioning (NCT02665065) and the CD45-astatine^211^ or 211^At-BC8-B10 antibody has a phase I/II trial ongoing to determine the optimal dose before allogeneic HSCT in patients with AML/ALL/MDS or mixed-phenotype acute leukemia (NCT03128034). As these targeted/non-genotoxic antibody-based conditioning targets progress, it will be important for the field to monitor how the reduction in host damage affects the GVHD penetrance after transplant.

### The Role of Cytokines in the GVH Response

One implication for the growth in targeted/non-genotoxic antibody-based conditioning regimens is that the normal cytokine storm fueled by the release of DAMPs from necrotic and pyroptotic cells will be diminished. While it is assumed these type of conditioning regimens will be better tolerated by the patient and reduce the number of NRM deaths, it is unclear how it will affect the frequency and severity of GVHD. In almost all model systems of allogeneic HSCT, the host is conditioned (by γ-irradiation) prior to transplant. This is both a requirement for a successful HSCT in murine, canine, porcine and non-human primate models and mimics what occurs in the clinic. Interestingly, many studies using the immunodeficient NSG mouse have continued this protocol of conditioning prior to transplant despite no longer being a requirement to study GVHD (75) (**Figure 1**). Since NSG mice are genetically pre-conditioned (i.e. lack all adaptive immune cells and have impaired innate immune responses), human cells can be adoptively transferred without prior conditioning (this is true for the study of human T cell responses though we acknowledge that for human HSPC engraftment, conditioning is almost always required) (47, 82).

In the studies performing xenogeneic transplantation in non-conditioned NSG mice, GVHD development can still occur even in the absence of host damage/cytokine storm. Thus, these limited number of studies suggests that inflammatory cytokines are not required for GVHD initiation though they undoubtedly influence the frequency, severity and pathology of the disease. For example, by studying which murine cytokines are cross-reactive with their cognate human receptors (in addition to which human cytokines are produced by activated human T cells), we may be able to investigate the role of individual cytokines in influencing disease pathology. A non-exhaustive list of murine cytokines and their cross-reactivity on human cells are highlighted in **Table 1**.

As mentioned above, the pathology of xenogeneic GVHD resembled that in the clinic though will a few important differences. The liver is one of the dominant organs affected during xenogeneic GVHD with skin GVHD occurring infrequently and mostly associated with the use of peripheral blood grafts (47, 48). From murine models, we know that liver and gastrointestinal tract GVHD (interestingly, GI GVHD is absent in non-conditioned NSG mice) is dominated by a TH_1_ response while skin GVHD is dominated by a TH_17_ response (21, 83, 84). The lineage commitment of T cells to the TH_1_ lineage is controlled by IL-12 while the TH_17_ lineage is controlled, in part, by IL-6 (70). Human T cells from xenogeneic mice are almost exclusively TH_1_ bias with only a small TH_17_ fraction observed and no TH_2_ population suggesting that the lineage commitment of the human T cells is skewed by some mechanism (47, 48, 85). Interestingly, while murine IL-12 is fully cross-reactive with the human IL-12 receptor, murine IL-6 and IL-4 are not cross-reactive, potentially explaining one mechanism by which human T cells become TH_1_ biased after xenotransplantation (56, 85–88).

The interferon family, specifically IFNγ, is arguably the most ubiquitous cytokine secreted by activated T cells and has been shown to have direct effects on GVHD pathology. In addition to being a feed-forward signal for T cell activation, IFNγ also has direct effects on HSPCs. While acute stimulation of human or murine HSPCs can result in robust myelopoiesis (e.g., in an infection), chronic IFNγ signaling results in the exhaustion and depletion of HSPCs progenitor populations (89–91). Specifically, IFNγ has been shown to sterically block the engagement of thrombopoietin (TPO) with its receptor c-MPL (90, 91). Transplantation with IFNγ-R1 KO bone marrow relieved this HSPC suppression in addition to suppressing T cell activation and GVHD (89). Less is known though about the role of type I interferons (IFNα/β) in GVHD. In several murine studies, type I interferons, specifically type I interferon receptor knockout and exogenous IFNα administration were able to prevent gastrointestinal tract GVHD by suppressing donor CD4^+^ T cell proliferation (92). These effects were also shown to be dependent on the activation of both MAVS and STING for full effect (93). Interestingly, the phosphorylation of STAT1, which also downstream of both type I and type II interferon receptors is generally considered to be pathogenic in regards to gastrointestinal GVHD (94–96). Phosphorylation of STAT1 in plasmacytoid dendritic cells (pDCs) causes them to drive TH_17_ differentiation with increases in both of these cell populations detected in the gastrointestinal biopsies of human GVHD patients (94). The presence of IL-22, secreted by TH_17_ cells in the gastrointestinal tract has also been shown to synergize with type I interferon signaling to enhance STAT1 phosphorylation and exacerbate GVHD (95). Additionally, it has been shown that the knock-out of STAT1 in donor CD4 T cells leads to the expansion of regulatory T cells, while knock-out of STAT1 in non-T cells leads to the expansion of STAT3+ pDCs and a reduction in GVHD severity (96, 97). Thus, it is currently unclear if the true role of type I interferons in GVHD is protective through the activation of MAVS and STING or harmful through the activation of STAT1. Type III interferons, such as IFNλ (IL-29), were recently shown to be protective against severe gastrointestinal GVHD in a mouse model of HSCT (98). Furthermore, pegylated IL-29 as able to enhance the survival of intestinal stem cells which protected against gastrointestinal damage (98). Despite the active roles for type I-III interferons in murine GVHD, only the type III interferons are cross-reactive between mice and humans, suggesting that while they may play an active role in GVHD pathology their role in GVHD development may be limited (99–101).

### Cytokine-Directed GVHD Prophylaxis in the Clinic

Despite the idea from non-conditioned xenogeneic transplant studies that cytokines may not be essential for GVHD development, they have been one of the most heavily investigated potential mechanisms for GVHD prevention and treatment. Surprisingly though, there has not yet been a clinical study identifying any of these inflammatory cytokines as biomarkers of the GVH response (102, 103). To date, the best biomarkers for HSCT are sST2 and REG3α, all of which are not secreted by immune cells (73, 74, 104). The release of sST2 is mediated by damaged endothelial stromal cells and REG3α is secreted by damaged intestinal epithelium cells. As such, while sST2 and REG3α have been used by the MAGIC consortium to predict NRM and SR-GVHD, they are representative markers of host damage and do not measure the degree of immunological activation from auto-reactive T cells in the host (73, 74, 104).

Overall, antibody and/or cytokine regimens for the treatment and/or prevention of GVHD have been met with mixed results. Cytokine therapies involving IL-1RA, IL-2 or an IL-1 decoy receptor have all failed to show efficacy in large phase III clinical trials (105–107). Antibodies against CD25 or TNFα have also failed to enhance the treatment of SR-GVHD compared to best available treatments (108–110). A promising antibody therapy discovered so far is tocilizumab, a humanized monoclonal antibody against the IL-6R. In several phase I/II clinical trial, tocilizumab showed efficacy in treating SR-GVHD, chronic GVHD and lowering the overall incidence of grade II-IV acute GVHD when administered early (111–113). Though in a more recent phase III randomized, double-blind trial (ACTRN12614000266662), tocilizumab given at day -1 resulted in a non-significant trend in the reduction of grade II-IV aGVHD and no improvement in long-term survival (114).

One hypothesis as to why antibody-directed therapies have not yet shown promise in the treatment/prevention of GVHD is because GVHD is a multi-faceted disease, influenced by a variety of cytokines secreted after condition and that the blocking of just one pro-inflammatory cytokine isn’t sufficient for efficacy (115, 116). As a result, tyrosine-kinase-inhibitors (TKIs) are now involved in multiple different clinical trials involving GVHD (**Table 2**). TKIs benefit from being able to suppress the signaling of multiple different cytokines at once through the inhibition of the JAK-STAT pathway (115, 116). In this mechanism, TKIs benefit from their broad suppressive profile though as a result, they have also been shown to have more adverse-events and a shorter half-life that antibody based therapies (115).

**Table 2:** JAK usage among the common cytokine receptor families.

Cytokine Receptor Family	Cytokines Affected	JAK Usage
**Type I Cytokine Receptors**		
Common y Chain (CD132)	IL-2, IL-4, IL-7, IL-9, IL-13, IL-15, IL-21	JAK1, JAK3
Common Chain β (CD131)	IL-3, IL-5, GM-CSF (CSF2), EPO, TPO	JAK2
gp130 (CD130)	IL6, IL-11, IL-12, IL-23, IL-27, LIF, OSM	JAK1, JAK2, TYK2
**Type II Cytokine Receptors**		
Interferon αβ Receptor	IFNα/β	JAK1, TYK2
Interferon γ Receptor	IFNγ	JAK1, JAK2
Type Ill Interferons	Type IIIIFN	JAK1, TYK2
IL-10 Receptor	IL-10, IL-20, IL-22, IL-28	JAK1
**Other Cellular Receptors**		
BCR	B-cells	BTK

The primary targets of TKIs used for GVHD are JAK1, JAK2 and BTK (115). While there are subtle differences in their use, JAK1/JAK2 broadly mediate the signaling of >20 cytokine receptors and BCR signaling in the case of BTK. This is due to the shared use of common signaling domains among cytokine receptors (115) (**Table 2**). While there are multiple TKIs FDA approved for a variety of diseases, there are only two currently FDA approved for GVHD related treatment. Ruxolitinib is a JAK1/2 inhibitor and the focus of the ongoing REACH trials (117). Recently, ruxolitinib was FDA approved for the treatment of SR-GVHD, the first new drug for SR-GVHD in the last 30 years after showing efficacy in a phase III trial (REACH II) (118, 119). Ruxolitinib is currently now in a phase III trial for the treatment of chronic GVHD (cGVHD) (REACH III). Another TKI, ibrutinib, which targets BTK, has already been approved for the treatment of cGVHD (120). Future studies comparing both ruxolitinib and ibrutinib in the treatment of cGVHD will yield important insights into redundant and non-redundant tyrosine kinase signaling during disease pathogenesis. Other TKIs in clinical trials include the selective JAK2 inhibitor, pacritinib, which is currently in a phase II clinical trials (NCT02891603). The authors reported that the pacritinib/sirolimus/tacrolimus prophylactic regimen was safe with its efficacy in preventing grade II-IV GVHD the subject of the phase II trial (121, 122). Another JAK1/2 inhibitor, baricitinib, is also investigating its efficacy in cGVHD patients through an ongoing phase I/II clinical trial (NCT02759731). Fostamatinib is an inhibitor of SYK, a B cell specific tyrosine kinase similar to BTK and is also being investigated in a phase I trial for the treatment of cGVHD (NCT02611063). While many of these TKIs have shown promising results, the failure of the JAK1 inhibitor itacitinib to meet its primary endpoints in the treatment of SR-GVHD as part of the GRAVITAS-301 phase III trial highlights the need for additional studies into the specific roles each kinase has in mediating GVHD pathology (123).

From both the clinical trial data and xenogeneic transplant studies, it is clear that cytokines have a major impact on GVHD pathogenesis. Pro-inflammatory cytokines secreted by innate immune cells have been well-studied in directing T cell adaptive responses (e.g. IL-12 promoting TH_1_ and IL-6 promoting TH_17_) with the family of TKI taking advantage of the promiscuous use of shared JAK proteins to suppress a broad number of cytokine signals. One criticism of TKIs though is the inability to modulate which cytokines are affected. For example, STAT3 activation downstream of the IL-6R promotes TH_17_ differentiation but STAT5 activation is also known to promote T_reg_ development (70, 124, 125). For this reason, drugs such as the ROCK2 inhibitor (belumosudil or KD025), which recently completely a phase II study for the treatment of cGVHD and is under review for FDA approval, may be ahead of its time. Pre-clinical studies have showed that belumosudil inhibits TH_17_ differentiation and promotes T_reg_ development through the inhibition/activation of STAT3 and STAT5 respectively (126, 127). Lastly, it is important to note that to date, there has not been a cytokine-directed-antibody or TKI therapy that has shown efficacy in preventing the development of grade II-IV aGVHD in the clinic. While the field will continue to investigate these classes of drugs, past studies and xenogeneic transplant models suggest that the role of cytokines in GVHD pathogenesis may only be efficacious as treatments of established GVHD after clinical symptoms have arose.

## Stimulatory/Inhibitory Ligands/Receptors (Signal 2)

So far in the review, we have shown that TCR ligation with allogeneic and/or xenogeneic antigen presented by HLA peptides is essential for GVHD development. We have also highlighted studies suggesting that cytokines may not be essential for the initiation of GVHD (i.e. the reactivity to allogeneic/xenogeneic antigens) but most likely are integral components of pathology and the perpetuation of disease. This leads to our last section on the role of co-stimulatory and co-inhibitory ligands on GVHD pathogenesis.

### Co-Stimulatory/Inhibitory Signaling During Xenogeneic Transplantation

Co-stimulatory receptors are generally divided into two superfamilies’ based on their extracellular domains, the immunoglobulin superfamily (IgSF) and the TNF receptor superfamily (TNFRSF). Importantly, their signaling pathways rely on adaptor proteins (e.g. TRADD, TRAF, FADD), MAP kinase signaling (e.g. ERK, JNK, P38) and transcription factor activation that is distinct from both TCR and cytokine signaling pathways (Zap70/PI3K and JAK/STAT respectively) (70).

While the importance of co-stimulation is well-known in murine models of GVHD, the relative contribution of each receptor/ligand pair on human GVHD is not as clear (70, 124). While xenogeneic transplant studies are now well-established and have the ability to investigate the importance of each co-stimulatory protein, that research has been hindered by not knowing which co-stimulatory proteins are cross-reactive between species are which are not (**Table 1**). Since GVHD develops in non-conditioned NSG mice that lack a cytokine storm, we believe there must be a subset of co-stimulatory proteins that are in-fact cross-reactive. To date, the only known proteins with cross-species reactivity is human CD28 and CTLA-4 for murine CD80/86 (B7-1 and B7-2 respectively) (128) (**Table 1**). Interestingly, one study showed that the infusion of a CTLA-4-Ig fusion protein, a well-documented inhibitor of human T cell activation, at the time of xenogeneic transplantation could prevent GVHD from developing in NSG mice (44). Furthermore, infusion of the CTLA-4-Ig fusion protein at the onset of GVHD was also able to rescue a subset of mice from death (44).

Of the many co-stimulatory ligand/receptor pairs that have shown efficacy in murine models, only OX40 (CD134), CD40L (CD154) and ICOS (CD278) have been shown to be either upregulated or maintain a high level of expression on human T cells after xenogeneic transplantation (47, 129). Of these three, only ICOS has been studied directly for its efficacy to prevent GVHD in xenogeneic transplantation. In this study, an antibody directed against human ICOS was injected at the time of transplant and was able to prevent lethal GVHD in 60% of mice (compared to 100% lethality in the control mice). This study though also noted that they were unable to control GVHD when the ICOS antibody was injected at later time points (130, 131). As the use of xenogeneic transplantations continues to grow, future studies investigating the role of each co-stimulatory protein on human T cells will be essential in our understanding of human GVHD pathogenesis (**Figure 1**).

### Co-Stimulatory Protein Based GVHD Prophylaxis in the Clinic

Currently, the most promising agent for GVHD prevention is Abatacept, a CTLA4 fusion protein currently being used in several clinical trials (132). In one recent phase II trial (ABA2), Abatacept in combination with standard calcineurin inhibitor plus methotrexate prophylaxis reduced the incidence of grade III-IV GVHD from 14.8% to 6.8% in 8/8 matched URD as part of a randomized double-blind placebo-controlled arm (133). Additionally, this study reported a decrease from 30.2% to 2.3% grade III-IV GVHD in a smaller 7/8 matched URD population compared to a nonrandomized matched cohort (133). This trial, which used an Abatacept dosing schedule of day -1, +5, +14 and +28 is now being extended as part of the ABA3 trial (NCT04380740) where all patients will be given the same four doses of Abatacept treatment followed by randomization and either another four doses of Abatacept or placebo. A second ongoing single arm, multi-center phase II study, ASCENT, is investigating an eight dose Abatacept treatment on pediatric patients with serious non-malignant hematological diseases undergoing mismatched URD transplants (NCT03924401).

The only other co-stimulatory protein based GVHD prophylaxis treatment currently under investigation is BMS-986004, a CD40L blocking antibody that is currently in a phase 1/2 open label trial (NCT03605927). The aim of this trial is to determine the safety of intravenous injection of BMS-986004 every two weeks starting on day +13 in conjunction with tacrolimus and sirolimus based GVHD prophylaxis and determine the efficacy in preventing grade II-IV aGVHD. In summary, while co-stimulatory proteins have been well-studied in murine models of HSCT, they have been vastly understudied when it comes their efficacy on human T cells, either in xenogeneic transplant models or the clinic. While the field of TKI for GVHD is highly exciting, these early studies also suggest that harnessing the power of co-stimulatory/inhibitory receptors may be the optimal target for future novel GVHD prophylaxis targets.

## Conclusions

While murine and clinical investigations into GVHD will remain workhorses in the field, it is clear that humanized mouse models are becoming increasingly utilized and offer a unique model system to directly investigate human T cell biology. Xenogeneic transplantation has already provided us insights into the importance of TCR: MHC interactions, the necessity of pro-inflammatory cytokines and a novel tool to investigate the role of co-stimulatory ligands in mediating GVHD development (**Figure 1**).

In addition to the GVHD treatments discussed above targeting specific T cell activation signals, cellular therapies for GVHD benefit from being able to target multiple pathways at once. Cellular therapies like Treg and mesenchymal stromal cell (MSC) were both studied in xenogeneic transplant models before moving into late stage clinical trials (134, 135). While the suppressive mechanism(s) identified for each cellular therapy are distinct, one common theme is the secretion of anti-inflammatory cytokines (e.g. IL-10, IL-35, TGF-β and PGE2) with only a limited role identified for inhibitory ligand/receptors (134, 135). Phase I trials using these adoptive therapies have shown promising but mixed results, most likely due to the inefficiencies and irregularities involved with ex vivo expansion of these cells (NCT04678401) (136). The large success of these therapies in both mouse models and xenogeneic transplant studies though suggests that future GVHD therapies may benefit from actively promoting anti-inflammatory cytokine production in addition and/or instead of solely blocking pro-inflammatory cytokines with xenogeneic transplant models serving as an excellent test-bed for such studies.

Abatacept remains the best example of the role xenogeneic transplantations will have in the future as the CTLA-4-fusion protein was first tested in xenogeneic transplantation models before moving into the clinic, where it has now become a highly promising candidate for preventing GVHD though its role in treating established GVHD remains uncertain (132, 133). Additionally, xenogeneic transplant models have questioned the role of pro-inflammatory cytokines in the development of GVHD (47, 75). While it is clear that the degree of host damage influences GVHD frequency/severity, it is unclear if cytokines are responsible for mediating this causation (12, 75). This hypothesis is supported tangentially by both xenogeneic transplant and clinical data suggesting that there may be a temporal switch in the importance of co-stimulatory proteins and cytokines with their effect mediated early and late post-transplant respectively (47, 107, 108, 110, 114). Lastly, xenogeneic transplant models have revealed to the field the variability of human clonal T cell responses even among inbred NSG mice (48, 62, 63). While the possibility of developing a computational model capable of predicting xeno- or allo-reactivity against a defined set of HLA molecules remains daunting, it will most likely be completed first in xenogeneic transplant model systems before transitioning to the clinic.

Their remains a plethora of exciting research possibilities that are now feasible thanks to the development of xenogeneic transplant model systems. Their feasibility will only grow as we learn more about the cross-reactivity of specific cytokines and co-stimulatory ligand/receptor pairs. With the rapid increase in single-cell-RNA-sequencing (scRNA-seq) and TCR-sequencing capabilities, researchers will be able to delve deeper into the clonality and unique gene expression patterns associated with human T cell responses post-transplant as well as the importance of KIR typing/mismatches in GVHD and GVL (31, 72). Additionally, the addition of proteosome inhibitors such as ixazomib and bortezomib, both of which are being studied in the context of cGVHD, and the generation of NSG mice expressing human HLA alleles, may aid in our investigations into the nature of xeno-reactive antigens (137). The goal of developing xenogeneic transplant models was to offer researchers a model system capable of studying human immune responses and to serve as a bridge from murine studies to clinical trials. In the end, we believe xenogeneic transplant studies have met this need and will continue to advance the field of GVHD research in the decades to come.

## Author Contributions

NH drafted the manuscript and performed the literature search. MB and CC revised the manuscript. All authors contributed to the article and approved the submitted version.

## Funding

This work was supported in part by NIH/NCATS UL1-TR002373 (NH), the Cormac Pediatric Leukemia Fellowship (NH) and the Stem Cell and Regenerative Medicine Center Fellowship (NH). Additional funding includes NIH/NIAID 75N93021C00004 (MB), NIH/NHLBI U01-HL134764 (MB), DOD W81XWH2010669 (MB), St. Baldrick’s-Stand Up to Cancer Pediatric Dream Team Translational Research Grant SU2C-AACR-DT-27-17 (CC), American Cancer Society Research Scholar grant RSG-18-104-01-LIB (CC), NIH/NCI R01 CA215461 (CC) and the MACC Fund (CC). The St. Baldrick’s Foundation collaborates with Stand Up to Cancer. Stand Up to Cancer is a division of the Entertainment Industry Foundation. Research grants are administered by the American Association for Cancer Research, the scientific partner of SU2C. None of these funding sources had any input in the study design, analysis, manuscript preparation or decision to submit for publication.

## Author Disclaimer

The contents of this article do not necessarily reflect the views or policies of the Department of Health and Human Services, nor does mention of trade names, commercial products, or organizations imply endorsement by the US Government.

## Conflict of Interest

CC reports honorarium from Nektar Therapeutics and Novartis, who had no input in the study design, analysis, manuscript preparation or decision to submit for publication. MB is a consultant for Taconic Biosciences.

The remaining author declares that the research was conducted in the absence of any commercial or financial relationships that could be construed as a potential conflict of interest.

## Publisher’s Note

All claims expressed in this article are solely those of the authors and do not necessarily represent those of their affiliated organizations, or those of the publisher, the editors and the reviewers. Any product that may be evaluated in this article, or claim that may be made by its manufacturer, is not guaranteed or endorsed by the publisher.
